# Impact of the COVID-19 pandemic on cardiovascular mortality and contrast analysis within subgroups

**DOI:** 10.3389/fcvm.2024.1279890

**Published:** 2024-02-07

**Authors:** Shoufang Song, Chen Guo, Ruiyun Wu, Hong Zhao, Qiang Li, Jia-hao Dou, Fan-shun Guo, Jin Wei

**Affiliations:** Department of Cardiology, The Second Affiliated Hospital of Xi'an Jiaotong University, Shaanxi, Xi'an, China

**Keywords:** COVID- 19, cardiovascular diseases, mortality, predictive analysis, disparity

## Abstract

**Background:**

An increase in deaths has been perceived during the pandemic, which cannot be explained only by COVID-19. The actual number of deaths far exceeds the recorded data on deaths directly related to SARS-CoV-2 infection. Data from early and short-lived pandemic studies show a dramatic shift in cardiovascular mortality. Grounded in the post-pandemic era, macroscopic big data on cardiovascular mortality during the pandemic need to be further reviewed and studied, which is crucial for cardiovascular disease prevention and control.

**Methods:**

We retrieved and collected data associated with cardiovascular disease mortality from the National Vital Statistic System from the Center for Disease Control and Prevention Wide-Ranging Online Data for Epidemiologic Research (CDC WONDER) platform based on the ICD-10 codes. We applied regression analysis to characterize overall cardiovascular disease mortality trends from 2010 to 2023 and built a time series model to predict mortality for 2020–2023 based on mortality data from 2010 to 2019 in order to affirm the existence of the excess deaths by evaluating observed vs. predicted mortality. We also conducted subgroup analyses by sex, age and race/ethnicity for the purpose of obtaining more specific sociodemographic information.

**Results:**

All-cause age-standardised mortality rates (ASMRs) for CVD dramatically increased between 2019 and 2021[annual percentage change (APC) 11.27%, *p* < 0.01], and then decreased in the following 2021–2023(APC: −7.0%, *p* < 0.01). Subgroup analyses found that the ASMR change was most pronounced in Alaska Indians/Native American people (APC: 16.5% in 2019–2021, −12.5% in 2021–2023, both *p* < 0.01), Hispanics (APC: 12.1% in 2019–2021, −12.2% in 2021–2023, both *p* < 0.05) and non-Hispanic Black people (APC:11.8% in 2019–2021, −10.3% in 2021–2023, both *p* < 0.01)whether during the increasing or declining phase. Similarly, the ASMR change was particularly dramatic for the 25–44 age group (APC:19.8% in 2019–2021, −15.4% in 2021–2023, both *p* < 0.01) and males (APC: 11.5% in 2019–2021, −7.6% in 2021–2023, both *p* < 0.01). By the end of 2023, the proportion of COVID-related excess death remained high among the elderly (22.4%), males (42.8%) and Alaska Indians/Native American people(39.7%). In addition, we did not find the presence of excess deaths in the young (25–44) and middle-aged cohort (45–64) in 2023, while excess deaths remained persistent in the elderly.

**Conclusions:**

All-cause ASMRs for CVD increased notably during the initial two years of the COVID-19 pandemic and then witnessed a decline in 2021–2023. The cohorts (the young, males and minorities) with the steepest rise in mortality decreased at the fastest rate instead. Previous initiatives to promote cardiovascular health were effective, but further research on cardiovascular healthcare for the elderly and racial disparities should be attached to priority considering the presence of sociodemographic differences in CVD death.

## Introduction

The Covid-19 pandemic placed unprecedented stress on the world's healthcare systems, and the pandemic severely disrupted medical care for U.S. patients with cardiovascular disease (CVD) and exacerbated cardiovascular-related risk factors (1). Existing short-term studies have shown dramatic changes in mortality among U.S. patients with CVD during the pandemic (2) and the pandemic with persistent threat may have long-term effects on patients with CVD, making it particularly important to review and analyze trends in cardiovascular diseases mortality with a holistic perspective in the post-pandemic era. This initiative is bound to provide effective information to health care providers and public health departments in order to mitigate the long-term effects of this pandemic.

However, limited data are available on overall changes in CVD mortality for the long-term period after the outbreak of this pandemic, including mortality data characterized by age, sex, and race (3). We therefore queried the U.S. Centers for Disease Control and Prevention (CDC) mortality dataset to characterize and predict trends in CVD mortality in order to expand from prior studies and understand the impact of the pandemic on these trends. We also explored whether this pandemic disproportionately affected CVD mortality in different age, gender and racial subgroups.

## Methods

### Data retrieval

This was a retrospective study using the data obtained from the National Vital Statistics System (NVSS) dataset through the Center for Disease Control and Prevention Wide-Ranging Online Data for Epidemiologic Research (CDC WONDER) website. The CDC WONDER dataset is publicly available and it is relatively easy to gain access and request data from it (4). Each record in the database represents the death data of one deceased person, which includes age, sex, race and ethnicity, and cause of death (5).

### Inclusion and exclusion criteria

The retrieval of death data in the database was carried out according to a variety of conditional logics, and after the preliminary research, we chose the following inclusion conditions for the study subjects. Ultimately, we chose to include data on cardiovascular disease-related deaths between January 1, 2010, and December 31, 2023, among adults aged no less than 25 years. As for the retrieval of causes of death, we opted International Statistical Classiﬁcation of Diseases, Tenth Revision (ICD-10) codes to identify the cause of death by CVD(I00-I99,diseases of the circulatory system) (6). Meanwhile, the corresponding ICD-10 code for COVID-19 was recognized as U07.1 which was classified under the codes for emergency use.

### Statistical analysis

The data ultimately applied directly to the statistical analysis included year and corresponding mortality. We finally chose to extract the age-standardized mortality rates(ASMRs, per 100,000 persons) for statistical analysis which were developed using the age structure (25–85 + years) from the 2000 USA Census Standard Population and the direct standardisation method (7).

First of all, we conducted joinpoint regression analysis to determine the nationwide trend of mortality in people with CVD (8). The overall trend is composed of one or several segments divided by corresponding joinpoint(s). Then we introduced an important parameter called the annual percentage change (APC) along with Monte Carlo Permutation test to determine whether the overall trend was best depicted by one or more segments (9). The positive/negative and absolute values of APCs represent the direction and extent of the trend, respectively (10).

Next, we constructed classical time series models(Autoregressive Integrated Moving Average Model, ARIMA) based on mortality data from 2010 to 2019 to determine predicted mortality rates for 2020–2023 and compared them to observed mortality rates from the dataset (11). After the above comparative analysis, we identified and assessed the excess mortality. We also further explored the COVID-19 related portion of the excess deaths described above, which refered to the portion of the excess deaths that might be explained by COVID-19.

Additionally, We divided the study subjects into several subgroups: age (25–44, 45–64, and ≥65 years), sex (male and female), race/ethnicity (Hispanics, non-Hispanic White people, non-Hispanic Black people, non-Hispanic Asian people and non-Hispanic American Indian/Alaska Native people) (12). We expect that the subgroup analysis could provide more detailed and precise information on mortality.

COVID-related deaths are defined as the portion of the death certificate in which COVID-19 is listed as the primary cause of death. All-cause mortality is defined as mortality from any cause (whether related to cardiovascular disease or not). CVD-related mortality is mortality due to complications of cardiovascular disease as the main cause of death. As for overall analysis, we calculated mortality rates for both (13). However, as for subgruop analysis, we preferred to use all-cause mortality which leads to more holistic evaluation effectiveness (13).

Statistical analysis was performed using the National Cancer Institute's joinpoint regression (Joinpoint Trend Analysis Software version 4.9.1.0; National Cancer Institute, Bethesda, MD) and IBM SPSS Statistics version 26.0. A 2-sided *p* value with the threshold of significance at 0.05 was used.

## Results

### Impact of COVID-19 pandemic on ASMRs for CVD

#### Overall analysis for all-cause ASMR

**Figure 1:**
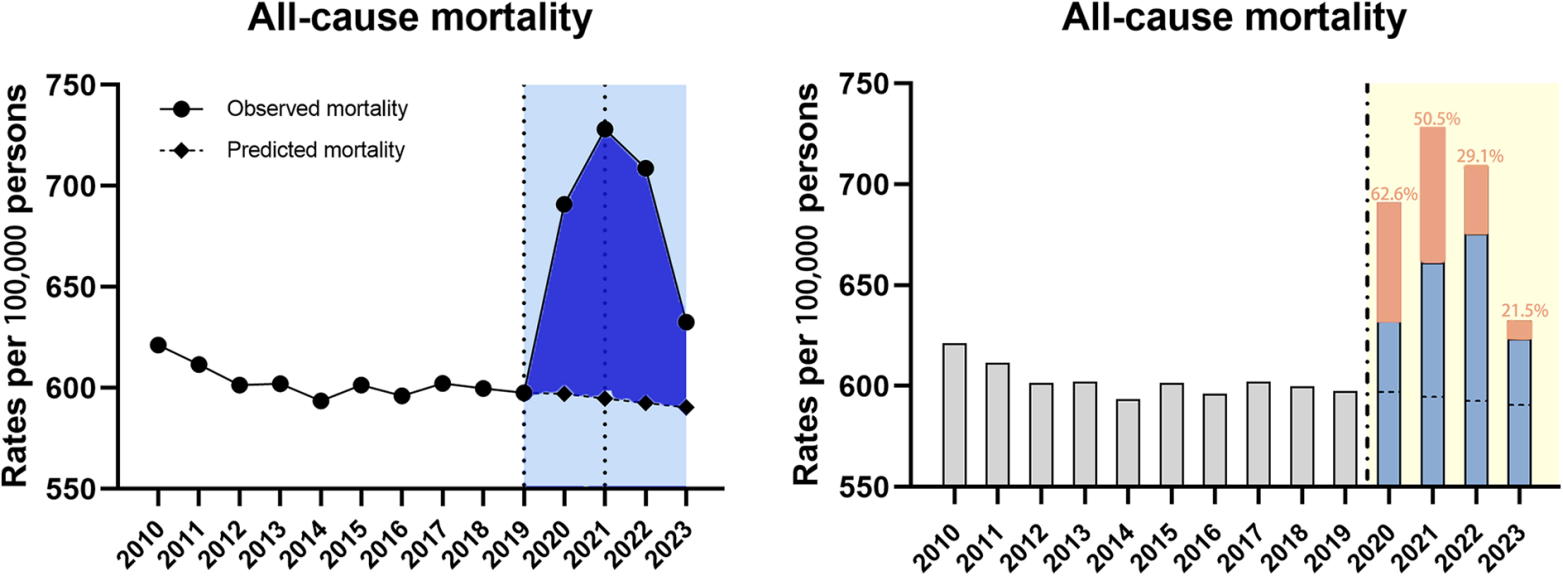
Temporal trends of all-cause mortality and excess deaths for CVD during the COVID-19 pandemic. Dashed horizontal line: predicted value; Blue bars: non-COVID related; Orange bars: COVID-related, with the proportion in the excess deaths.

After joinpoint regression analysis, the overall trend was divided into three segments bounded by 2019 and 2021 with the average APC of 1.1% (95% CI 0.2–2.0) for 2010–2023 (Table 1). As for the trend segment analysis, we found accelerated APC from −0.2% for 2010–2019 to 11.3% (95% CI 7.8–14.8, *P* < 0.001) for 2019–2021 but the following declining trend with the APC of −7.0% (95% CI −10.1 to −3.8, *P* < 0.05) for 2021–2023 (Table 1). Similarily, we also found the observed ASMRs in 2020–2023 were all much higher than the predicted values demonstrating significant excess mortality, which was was more dramatic in 2021 and then showed a declining trend (Figure 1). Notably, more than half of the excess deaths in the first two years of the pandemic (62.6% in 2020, 50.5% in 2021) were associated with COVID-19, and this proportion moderated in 2022–2023 (29.1% in 2022, 21.5% in 2023) (Figure 1).

**Table 1:** ASMR and APC in mortality in U.S. adults with CVD, by all-cause mortality and CVD-related mortality, 2010-2023.

	Age-standardised rates per 100,000			APC (95%CI)	Curve segment		*P* value
	2010 (pre-epidemic period)	2021	2023	2010–2023	Year	APC (95% CI)	
All-cause mortality	621.18	728.08	622.04	1.1 (0.2–2.0)	2010–2019	−0.19 (−0.5 to 0.1)	0.204
					2019–2021	11.27 (7.8–14.8)	<0.001
					2021–2023	−7.0 (−10.1 to −3.8)	0.002
CVD-related mortality	363.02	345.96	359.58	−0.3 (−1.3 to 0.6)	2010–2019	−0.8 (−1.2 to −0.4)	0.001
					2019–2021	4.34 (0.5–8.3)	0.031
					2021–2023	−2.7 (−4.9 to −0.3)	0.034

#### Overall analysis for CVD-related ASMR

**Figure 2:**
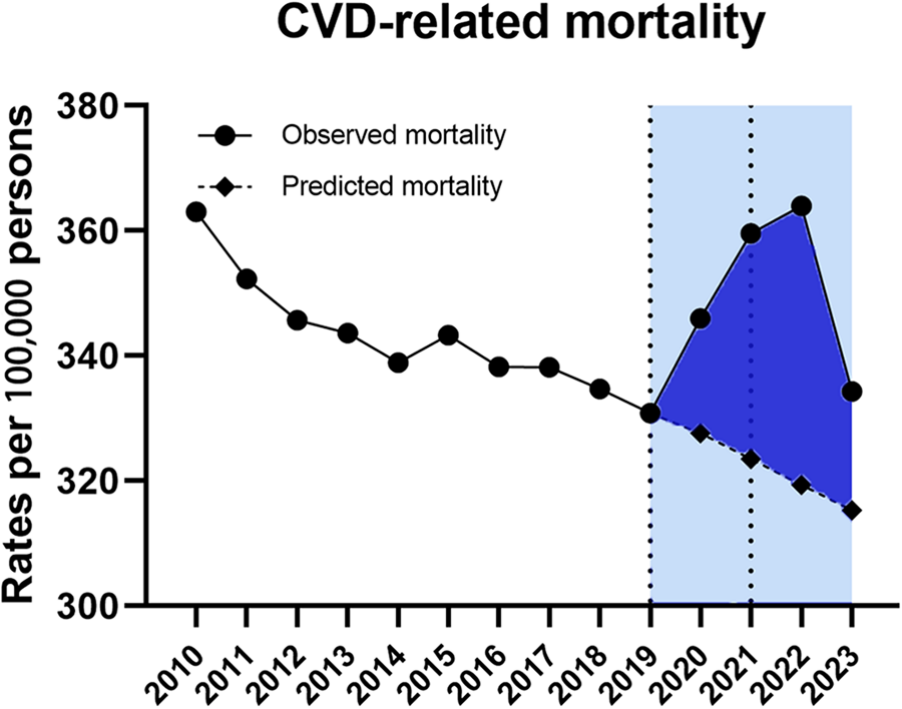
Temporal trend of CVD-related mortality during the COVID-19 pandemic.

Data for CVD-related ASMRs followed the similar trend, which was also divided into three segments bounded by 2019 and 2021 (Table 1). On trend segment analysis, we found increased APC from −0.8% (95% CI −1.2–0.4) for 2010–2019 to 4.3% (95% CI 0.5–8.3) for 2019–2021 which was followed by a declining trend with the APC of −2.7% (95%CI −4.9 to −0.3, *P* < 0.05) for 2021–2023 (Table 1). As a result, the observed ASMRs (345.96 in 2020, 359.58 in 2021, 363.99 in 2022 and 334.31 in 2023) were much higher than the predicted values of 327.26 (95% CI 577.57–607.86) for 2020, 323.69 (95% CI 577.57–607.86) for 2021, 319.38 (95% CI 288.56–350.20) for 2022 and 315.27 (95% CI 271.54–359.01) for 2023 showing the excess death for CVD-related mortality (Figure 2).

#### Subgroup analysis for all-cause ASMR

##### By age group

**Figure 3:**
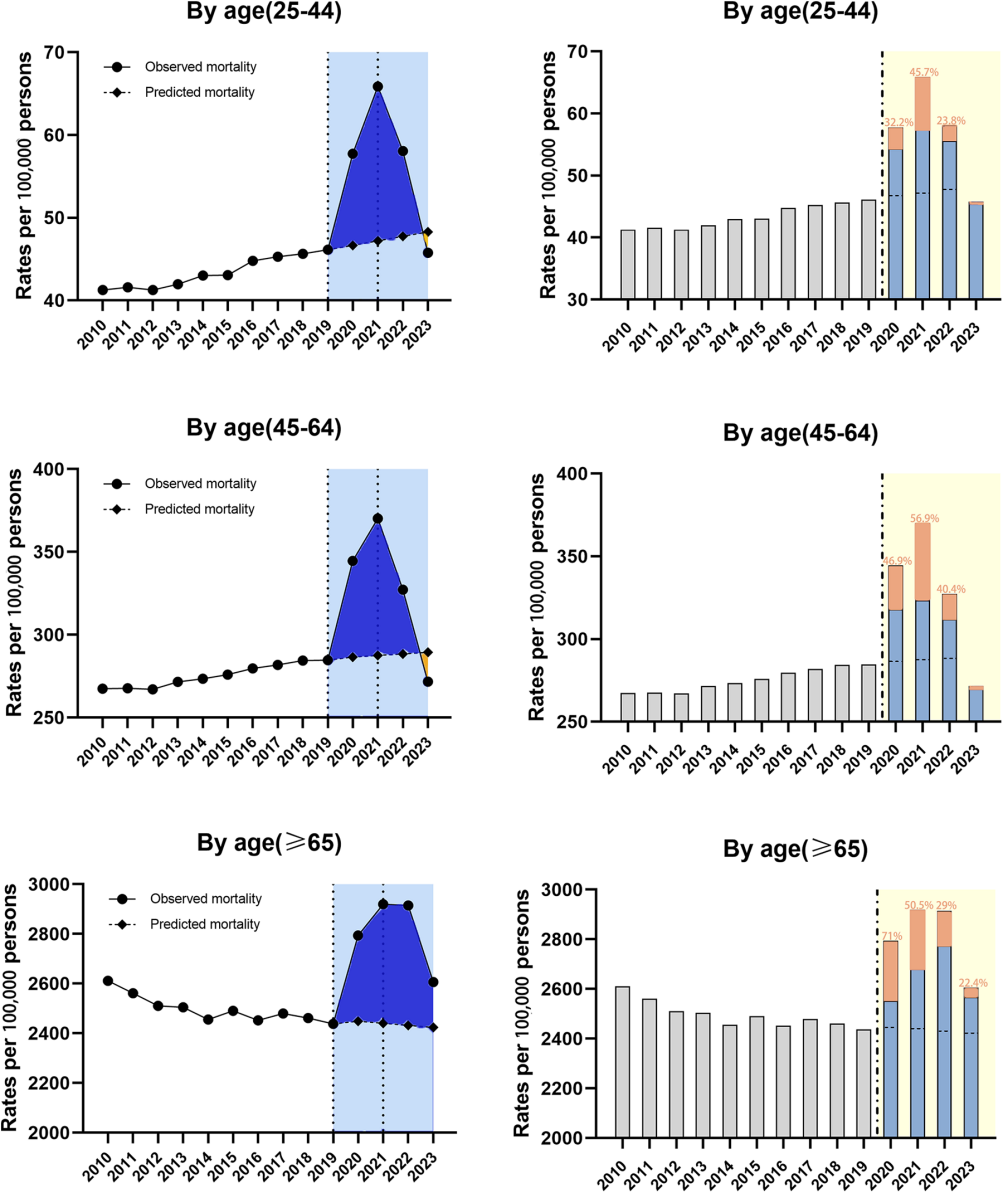
Temporal trends of all-cause mortality and excess deaths for CVD during the COVID-19 pandemic, by age. Dashed horizontal line: predicted value; Blue bars: non-COVID related; Orange bars: COVID-related, with the proportion in the excess deaths.

The highest ASMR was seen in the elderly group throughout the study period. All the overall trends of three subgroups were described by three segments bounded by 2019 and 2021 based on the joinpoint regression analysis (Table 2). Subgroup analysis by age found ASMR rise across the entire study period 2010–2023 among all the three groups with the average APC 2.9% for 25–44, 1.8% for 45–64 and 0.9% for ≥65 (Table 2). During the pre-epidemic period, there was a downward trend in mortality among the elderly, while the mortality in the younger and middle-aged groups steadily increased. What's more, the 2019–2021 APCs for ASMR showed significant rise in all age groups with the largest change for 25–44 (19.8%, 95% CI 16.5–23.8, *P* < 0.001) compared to 45–64 (14.5%, 95% CI 12.7–16.2, *P* < 0.001) and ≥65 (11.6%, 95% CI 6.0–17.6, *P* < 0.05) (Table 2). However, the increasing trend in 2019–2021 was followed by a decline in 2021–2023 in all groups with the steepest change for 25–44 (−15.4%, 95%CI −18.7 to −12.1, *P* < 0.001) compared to 45–64 (−14.4%, 95% CI −16.0 to −12.8, *P* < 0.001) and ≥65 (−5.0%, 95%CI −8.6 to −1.4, *P* < 0.05) (Table 2). Observed mortality rates in 2020–2022 were higher than the predicted mortality rates for all three subgroups, while we did not observe excess deaths in the young and middle-aged group in 2023(predicted mortality higher than the actual rate). Of note, the portion of excess deaths associated with COVID-19 was higher in the middle-aged and older age groups compared to the young group during the study period (Figure 3).

**Table 2:** All-cause ASMR and APC in mortality in U.S. adults with CVD, by age subgroup, 2010–2023.

	Age-standardised rates per 100,000			APC (95%CI)	Curve segment		*P* value
	2010 (pre-epidemic period)	2021	2023	2010–2023	Year	APC (95% CI)	
25–44	41.27	65.87	45.77	2.9 (1.3–4.6)	2010–2019	1.5 (1.2–1.9)	<0.001
					2019–2021	19.8 (16.0–23.8)	<0.001
					2021–2023	−15.4 (−18.7 to −12.1)	<0.001
45–64	267.45	370.14	267.78	1.8 (0.4–3.1)	2010–2019	1.0 (0.8–1.1)	<0.001
					2019–2021	14.5 (12.7–16.2)	<0.001
					2021–2023	−14.4 (−16.0 to −12.8)	<0.001
≥65	2,610.99	2,919.43	2,606.01	0.9 (0–1.7)	2010–2019	−0.5 (−0.9 to −0.2)	0.012
					2019–2021	11.6 (6.0–17.6)	0.002
					2021–2023	−5.0 (−8.6 to −1.4)	0.016

##### By sex

**Figure 4:**
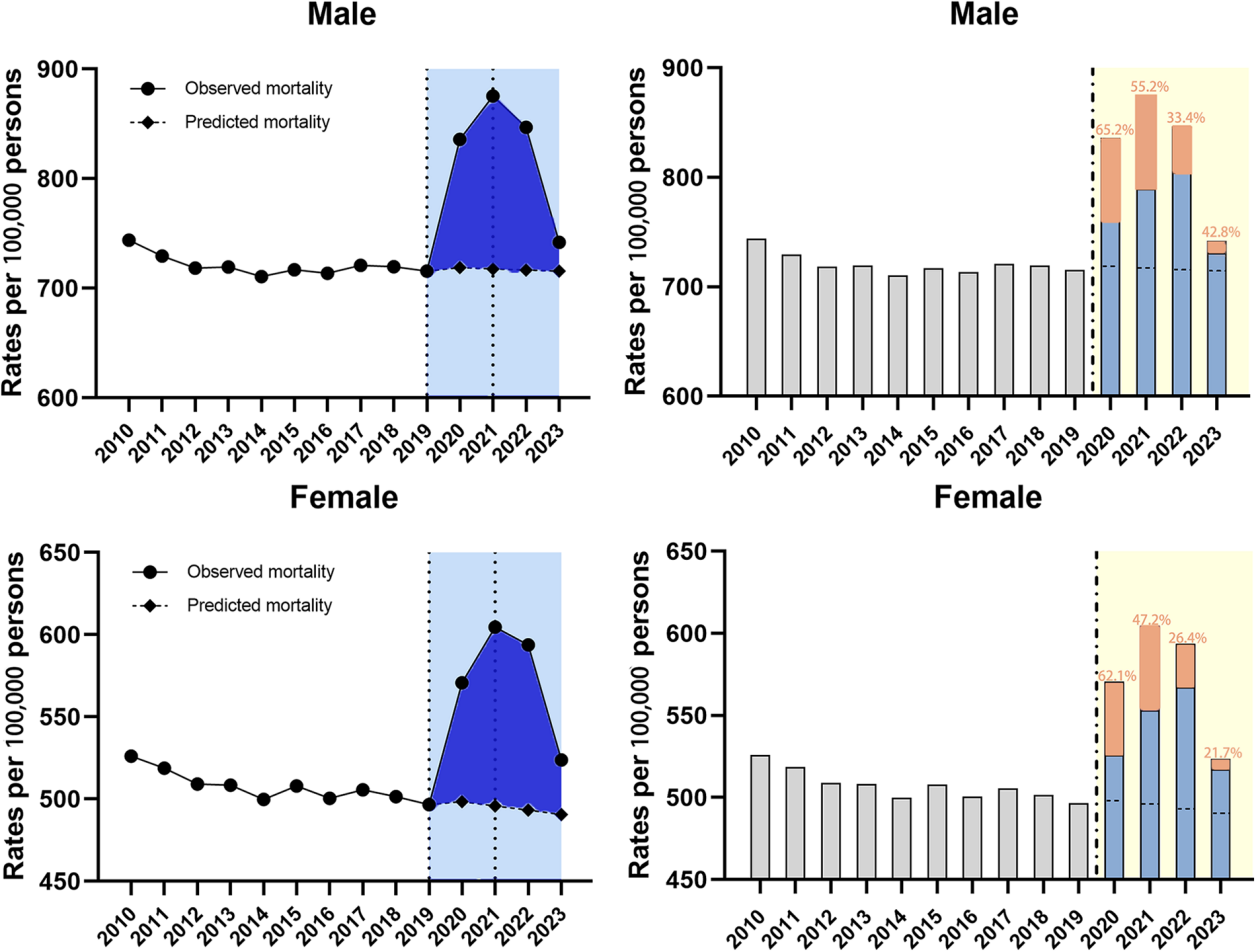
Temporal trends of all-cause mortality and excess deaths for CVD during the COVID-19 pandemic, by sex. Dashed horizontal line: predicted value; Blue bars: non-COVID related; Orange bars: COVID-related, with the proportion in the excess deaths.

Subgroup analysis by sex found that the mortality burden was consistently higher for males than for females. Regression analysis showed that overall trend was divided into three segments bounded by 2019 and 2021 (Table 3). Significant ASMRs rise can be found during the 2019–2021 period in both subgroups (Table 3, both *P* < 0.001). Then the mortality rate noticed a declining trend in both groups, as evidenced by the APC of −7.6% (95% CI −10.7 to −4.3, *P* < 0.05) for males and −6.2% (95% CI −9.4 to −2.9, *P* < 0.05) for females in 2021–2023 (Table 3). As a result, the observed ASMRs were signiﬁcantly higher than expected rates for both males and females showing the existence of excess deaths. Furthermore, the COVID-related component of excess deaths was larger in the male cohort during the study period (Figure 4).

**Table 3:** All-cause ASMR and APC in mortality in U.S. adults with CVD, by sex subgroup, 2010–2023.

	Age-standardised rates per 100,000			APC (95%CI)	Curve segment		*P* value
	2010 (pre-epidemic period)	2021	2023	2010–2023	Year	APC (95% CI)	
Male	743.82	875.33	741.8	1.1 (0.2–2.1)	2010–2019	−0.1 (−0.5 to 0.2)	0.477
					2019–2021	11.5 (7.9–15.2)	<0.001
					2021–2023	−7.6 (−10.7 to −4.3)	0.001
Female	526.06	604.52	523.67	0.9 (0–1.8)	2010–2019	−0.4 (−0.7 to −0.1)	0.023
					2019–2021	10.9 (7.4–14.4)	<0.001
					2021–2023	−6.2 (−9.4 to −2.9)	0.004

##### By race/ethnicity

**Figure 5:**
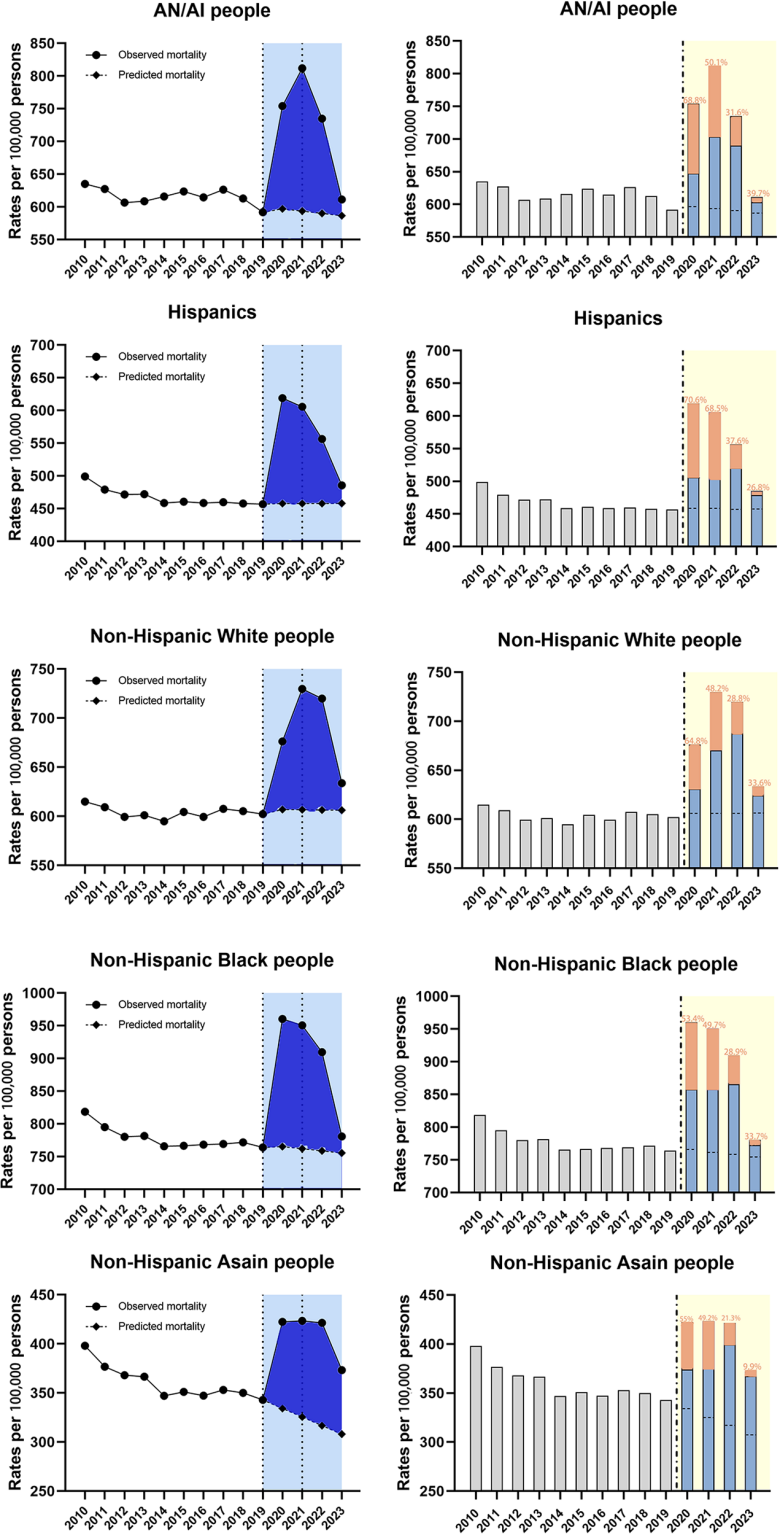
Temporal trends of all-cause mortality and excess deaths for CVD during the COVID-19 pandemic, by race/ethnicity. Dashed horizontal line: predicted value; Blue bars: non-COVID related; Orange bars: COVID-related, with the proportion in the excess deaths.

During the entire study period, mortality trends for several racial subgroups were divided into three or four segments based on the regression analysis (Table 4). Besides, the most dramatic APC rise during the 2019–2021 period was in American Indians/Alaska Native people (16.5%, 95% CI 12.0–21.2, *p* < 0.001) followed by non-Hispanic Black people(11.8%, 95% CI 9.0–14.6, *p* < 0.001), and non-Hispanic Asian people (11.1%, 95% CI 6.6–15.8, p < 0.05), with the shortest rise during the same period in non-Hispanic White people (10.4%, 95% CI 7.1–13.8, *p* < 0.001) (Table 4). Meanwhile, the regression analysis found the declining trends during 2021–2023 among all the subgroups, with the most significant change of APC for American Indians/Alaska Native people (−12.5%, 95% CI −16.7 to −8.1, *p* < 0.05) (Table 4). For all racial subgroups, the observed mortality was higher than the predicted mortality showing the existence of excess deaths (Figure 5). Moreover, the highest proportion of COVID-related component of excess deaths during 2020–2021 was found among the Hispanics (70.6% in 2020, 68.5% in 2021) and AN/AI people (68.8% in 2020, 50.1% in 2021), and this proportion remained relatively high among AN/AI people (39.7%) and non-Hispanic Black people (33.7%) by the end of 2023 (Figure 5).

**Table 4:** All-cause ASMR and APC in mortality in U.S. adults with CVD, by race subgroup, 2010–2023.

	Age-standardised rates per 100,000			APC (95%CI)	Curve segment		*P* value
	2010 (pre-epidemic period)	2021	2023	2010–2023	Year	APC (95% CI)	
AN/AI people	635.21	811.94	611.33	1.4 (0–2.9)	2010–2019	−0.2 (−0.6 to 0.3)	0.462
					2019–2021	16.5 (12.0–21.2)	<0.001
					2021–2023	−12.5 (−16.7 to −8.1)	0.001
Hispanics	499.01	605.62	485.67	1.5 (0–3.2)	2010–2019	−1.0 (−3.4 to 1.4)	0.36
					2019–2021	12.1 (2.3–22.8)	0.021
					2021–2023	−12.2 (−15.7 to −8.5)	<0.001
Non-Hispanics White people	614.91	729.69	633.71	1.2 (0.3–2.0)	2010–2019	−0.0 (−0.4 to −0.3)	0.747
					2019–2021	10.4 (7.1–13.8)	<0.001
					2021–2023	−5.7 (−8.9 to −2.4)	0.006
Non-Hispanics Black people	818.72	950.59	780.94	1.1 (−0.1 to 2.3)	2010–2015	−1.3 (−2.0 to −0.6)	0.006
					2015–2019	1.1 (−0.3 to 2.6)	0.097
					2019–2021	11.8 (9.0–14.6)	<0.001
					2021–2023	−10.3 (−13.7 to −6.6)	0.001
Non-Hispanics Asian people	398.04	423.37	373.28	0.9 (−0.3 to 2.1)	2010–2014	−3.1 (−4.8 to −1.3)	0.009
					2014–2019	0.4 (−1.2 to 2.0)	0.537
					2019–2021	11.1 (6.6–15.8)	0.002
					2021–2023	−6.7 (−12.9 to −0.2)	0.046

## Discussion

In this study, we updated the evaluation on the impact of the COVID-19 pandemic on mortality in CVD patients by characterising the temporal trends in cardiovascular disease mortality from 2010 to 2023. First, the regression analysis showed a striking increase in both all-cause mortality and CVD-related mortality among CVD patients during 2019–2021, which was followed by a downward trend in mortality during 2021–2023. The mortality surge even reversed pre-pandemic trends in nearly all subgroups, most of which were not contributed by direct effect of COVID-19. Second, relevant APCs showed that the shift in mortality trends (whether increasing or subsequent declining trends) was more significant in the youngest group (25–44), males and minorities, respectively. Third, excess mortality was observed in all subgroups, which declined from 2021 but persisted in the vast majority of subgroups (except the young and middle-aged groups) until 2023. The percentage of COVID-related portion of excess deaths was in generally decreasing trend, but it remained high among the elderly, males and some minorities.

The increased CVD mortality caused by COVID-19 pandemic was well documented, which was reported from the initial phase of the pandemic to following one or two years. In the early months of the pandemic, studies showed a significant increase in deaths from CVD compared to the same period in 2019 (14). Subsequently, other studies comparing cardiovascular mortality rates before and after the pandemic found significant increase in mortality and excess deaths despite 2 years since the onset of the pandemic (6). A recent study demonstrated elevated cardiovascular deaths persisted until early 2022 by characterizing trends in excess CVD deaths (15). Our study expands from prior work by analyzing recent provisional data from the online database covering a longer time span, describing trends in mortality, comparing observed to predicted mortality rates, assessing the excess deaths and further stratifying by age, sex and race/ethnicity.

The increased excess CVD deaths during the pandemic was attributed to a combination of effects, whether direct or indirect factors. In one hand, SARS-CoV2 infection is closely associated with acute cardiovascular complications such as myocarditis, acute coronary syndrome and microvascular thrombosis, and may accelerate or activate the development of existing subclinical disease, which undoubtedly leads to myocardial injury in healthy people or patients with cardiovascular diseases (16). Survivors of SARS-CoV-2 infection are at increased long-term risk for various chronic cardiovascular diseases, with reports estimating a relative increase of approximately 55% in the risk of major adverse cardiovascular events over the next year (17). In addition, post-COVID-19 cardiac syndrome had been reported among COVID-19 survivors (18). SARS-CoV-2 targets multiple cells expressing ACE-2, which would lead to corresponding dysfunction and alteration of the cardiometabolic environment such as new onset diabetes and chronic kidney disease (19). In another hand, the indirect effects of the pandemic are far-reaching and cannot be ignored. In the initial phase of the epidemic, emergency medical care was usually directed towards the patients with acute infection, and non-essential medical services were suspended (20). Multinational studies have shown that all phases of health care for patients with cardiovascular disease (outpatient visits, admissions, diagnosis, and treatment) are affected to varying degrees during a pandemic, mainly in terms of decreased outpatient activity, fewer hospital admissions, delayed visits, worse admissions, and reduced and delayed diagnostic and treatment procedures (21). Besides, lockdown measures also brought about barriers and obstacles for seeking medical services. Relevant studies analyzed and summarized cardiovascular-related risk factors in the general population during the pandemic, showing a decrease in physical activity and an increase in sedentary behavior in all age groups during the epidemic lockdown (22). What's more, the increase in alcohol and tobacco intake and the deterioration in dietary structure also raised concerns (23). Such aggravated risk factors also induced adverse cardiovascular outcomes attributing to the increased mortality.

As time went on reports indicated that increased mortality could not be explained by COVID-19 alone and concerns arose about the effects of the pandemic on patients with non-communicable chronic diseases such as cardiovascular diseases (24). Similarly, our findings suggested that the non-COVID-directly related component also accounted for a certain proportion of excess deaths. Therefore, strategies and initiatives emerged to promote cardiovascular health during the pandemic. Professional cardiovascular associations and organizations issued several guidelines and consensus covering a wide range of topics such as emergency life support, diagnosis, treatment and cardiac care of CVD during the epidemic, and advice on rehabilitation of the cardiovascular sequelae of COVID-19 (25–27). Healthcare resource allocation was optimized and medical services for CVD were improved as well. By the end of 2020, emergency medical services in U.S. had recovered to some extent (28). A worldwide study coordinated by The International Atomic Energy Agency (IAEA) showed a 4% increase in cardiovascular testing in the U.S. in April 2021 compared with pre-pandemic baseline volumes (29). Application of telemedicine visits provided additional opportunities for cardiovascular health monitoring (30). Besides, one year into the pandemic, COVID-19 drug therapy and vaccines were approved (31, 32). These factors mentioned above may help to explain the declined trend in mortality from 2021 to 2023 based on the regression analysis in our study.

Furthermore, we also focused on the demographic comparisons. Regression analysis showed that the change of mortality was more dramatic in the younger cohort (25–44), with an APC of 19.8% in 2019–2021, almost twice that of the older cohort (≥65). The younger adults experienced the greatest relative increase in mortality during the initial period. Similar reports can be found in published literature as well. A retrospective study identified age related differences in CVD mortality trends over the first year of the pandemic in USA showing that adults aged 55 and younger experienced the largest increases in mortality (assessed by APC as well) vs. the older groups (33). Similarly, another study found the APC in deaths over the initial period during the pandemic in USA was largest for adults aged 25–44 years (34). There may be several explanations for these findings. First, younger cohorts may be more susceptible to pandemic indirect factors, as another study found that mortality trends in this age group were statistically associated with the strength of the intervention (35). Second, reduced physical activity during the intervention is perhaps more common in the young, who have to work from home compared to the elderly, which explains their sedentary lifestyles (36). However, sedentary lifestyles have a negative impact on cardiovascular outcomes. Third, adults in U.S. who are under Medicare age (generally under 65) have unequal access to health insurance, which may affect the access to health care and thus lead to an increase in mortality. Meanwhile, the elderly with cardiovascular diseases may actively seek outpatient care, whereas healthier young people may delay seeking care (37). In addition, the elderly still experienced the greatest absolute mortality and excess deaths remained persistent even in 2023 compared to the other two groups. Our findings imply that the government or health authorities may prudently reconsider about restrictions in the early stage of the pandemic, as well as health education for the young group. Meanwhile, health insurance policies should be altered in due course. However, considering the persistence of excess deaths among the elderly recently, concerns about long-term impacts of COVID-19 on these patient populations should be attached to higher priorities.

Overall mortality levels were higher for male patients than for females, with a slightly more obvious increasing trend in mortality for males than for females over the 2019–2021 period. Our findings parallel other studies that reported higher mortality in males (38, 39), and some factors may help explain this phenomenon. First of all, higher rates of CVD comorbidity in males may be associated with higher mortality rates, which is mentioned in published papers (40, 41). Next, sexual disparities in immune-inflammatory responses may affect the relation between CVD and COVID-19 risk and therefore lead to the higher mortality in males (42). Relevant studies have shown that estrogen and the X chromosome in women play a vital role in developing a more powerful response to infectious diseases and greater immune-mediated tissue repair (40). In addition, CVD and COVID-19-related inflammation/immune dysregulation might combine to increase risk associated with both diseases (43). Our findings on gender differences in mortality may encourage researchers to consider gender as a variable in research on the relationship between COVID-19 and CVD.

Moreover, our findings also demonstrated the diverse impact of the pandemic on different racial/ethnic groups. Non-Hispanic Black people mortality rates topped the list, while American Indian/Alaska Native people and non-Hispanic White people mortality rates were closer and in the middle of the pack. Hispanics had the forth highest death rate, what's more, non-Hispanic Asian people maintained the lowest level of mortality during the pandemic. In terms of the mortality trends, American Indian/Alaska Native people, Hispanics and non-Hispanic Black people had the top three steepest upward trend during pandemic according to the APCs in 2019–2021. However, Non-Hispanic white people, who are demographically predominant, had the slowest change in mortality trend. Racial disparities in cardiovascular disease mortality during the pandemic are notably discouraging. One rational explanation for this phenomenon concentrates on a higher prevalence of CVD comorbidities in minority populations, and avoidance of medical care may have greater impact on these populations (44). However, it may only play a small role compared to racism and health inequities. A study of the relationship between CVD mortality and socio-demographic factors during the COVID-19 pandemic, using the Socio-Vulnerability Index (SVI), further revealed this phenomenon (45). SVI refers to the sum of economic, demographic, and social factors that affect a community's risk exposure and ability to respond to hazardous events such as natural disasters (46). This study confirms the large overlap between black people communities and areas with high SVI indices and the significant correlation between SVI and CVD mortality is enhanced during the epidemic (46). The lower utilization of telemedicine by minorities and lower socioeconomic groups during the epidemic may also have increased the risk of adverse cardiovascular events (47). In addition, one recent meta-analysis found that the risk of infection remained elevated among ethnic minority groups beacuse of poor ventilation in homes and workplaces (48). That is, structural and social factors, such as housing and job insecurity or high poverty rates, were already contributing to the increased burden of CVD in specific communities prior to the pandemic, and the COVID-19 pandemic has largely exacerbated these social disadvantages (49). Recent study pointed that the previous barriers to vaccine readiness among minorities had been basically addressed (50). Nevertheless, racial disparities in healthcare access persisted with minority populations and sociodemographic disparities in telemedicine use was increasing (51, 52). The declined trend of mortality in 2021–2023 can be attributed to combined forces such as policies shift, immunity and re-distribution of healthcare resources for CVD, however, further assessment of these disparities and the development of strategies cannot be ignored.

The strength of our study was that we used the regression analysis and predictive models to demonstrate changes in mortality trends and predict mortality rates during the pandemic separately. We also devided the study population into subgroups in order to provide more detailed demographic characteristics. The study also had some limitations. We did not focus on non-CVD-related deaths and data for specific cardiovascular diseases were not included in our study. The death dataset we used is more readily available and easier to retrieve and organize data. In addition, the dataset has a broader coverage and offers a variety of categorical search options, providing more detailed information on deaths while minimizing selection bias. However, such large database may incorrectly code the cause of death, especially in the early stage of the pandemic. In addition, the database lack important clinically relevant information such as nursing procedures, surgical treatments, etc.

## Conclusions

Our study provided more detailed information on mortality trends for CVD during the pandemic in U.S. The mortality significantly increased in the initial two years of the pandemic, which was followed by a decline in 2022–2023. Further subgroup analyses found that mortality trends were more dramatic in the young, male and minority populations. By the end of 2023, excess death remained persistent in nearly all groups except the young and middle-aged cohorts. Considering the necessity for the research on long-term impacts of COVID-19, further research on cardiovascular healthcare for the elderly and racial disparities should continue to be emphasized. We are also committed to providing policymakers and healthcare executives with information to optimize resource allocation in the post-epidemic era in order to improve the resilience to similar events in the future.

## Data Availability

Publicly available datasets were analyzed in this study. This data can be found here: https://wonder.cdc.gov/mcd.html.
